# Diagnostic accuracy of clinical tools for assessment of acute stroke: a systematic review

**DOI:** 10.1186/s12873-019-0262-1

**Published:** 2019-09-04

**Authors:** Daria Antipova, Leila Eadie, Ashish Macaden, Philip Wilson

**Affiliations:** 1 Centre for Rural Health, University of Aberdeen, Old Perth Road, Inverness, IV2 3JH UK; 2 Department of Stroke and Rehabilitation, Raigmore Hospital, NHS Highland, Inverness, IV2 3UJ UK

**Keywords:** Acute cerebral ischaemia, Clinical prediction rules, Emergency care, Intracerebral haemorrhage, Large vessel occlusion, Recanalization, Stroke, Scoring methods, Thrombectomy, Thrombolysis

## Abstract

**Introduction:**

Recanalisation therapy in acute ischaemic stroke is highly time-sensitive, and requires early identification of eligible patients to ensure better outcomes. Thus, a number of clinical assessment tools have been developed and this review examines their diagnostic capabilities.

**Methods:**

Diagnostic performance of currently available clinical tools for identification of acute ischaemic and haemorrhagic strokes and stroke mimicking conditions was reviewed. A systematic search of the literature published in 2015–2018 was conducted using PubMed, EMBASE, Scopus and The Cochrane Library. Prehospital and in-hospital studies with a minimum sample size of 300 patients reporting diagnostic accuracy were selected.

**Results:**

Twenty-five articles were included. Cortical signs (gaze deviation, aphasia and neglect) were shown to be significant indicators of large vessel occlusion (LVO). Sensitivity values for selecting subjects with LVO ranged from 23 to 99% whereas specificity was 24 to 97%. Clinical tools, such as FAST-ED, NIHSS, and RACE incorporating cortical signs as well as motor dysfunction demonstrated the best diagnostic accuracy. Tools for identification of stroke mimics showed sensitivity varying from 44 to 91%, and specificity of 27 to 98% with the best diagnostic performance demonstrated by FABS (90% sensitivity, 91% specificity). Hypertension and younger age predicted intracerebral haemorrhage whereas history of atrial fibrillation and diabetes were associated with ischaemia. There was a variation in approach used to establish the definitive diagnosis. Blinding of the index test assessment was not specified in about 50% of included studies.

**Conclusions:**

A wide range of clinical assessment tools for selecting subjects with acute stroke has been developed in recent years. Assessment of both cortical and motor function using RACE, FAST-ED and NIHSS showed the best diagnostic accuracy values for selecting subjects with LVO. There were limited data on clinical tools that can be used to differentiate between acute ischaemia and haemorrhage. Diagnostic accuracy appeared to be modest for distinguishing between acute stroke and stroke mimics with optimal diagnostic performance demonstrated by the FABS tool. Further prehospital research is required to improve the diagnostic utility of clinical assessments with possible application of a two-step clinical assessment or involvement of simple brain imaging, such as transcranial ultrasonography.

**Electronic supplementary material:**

The online version of this article (10.1186/s12873-019-0262-1) contains supplementary material, which is available to authorized users.

## Rationale

Patients with acute stroke should have access to rapid assessment and early intervention with specialist care for optimal outcomes. Acute ischaemic stroke caused by a large vessel occlusion (LVO) is associated with high mortality rate of 80% [1] and can be optimally managed with intravenous (IV) thrombolysis followed by mechanical thrombectomy (MT). While IV thrombolysis can currently be provided in many general hospitals, MT can only be performed in specialised centres with neurointerventional facilities.

Recanalization therapy must be delivered within the first hours after symptom onset to improve functional outcome [2, 3]. This requires a reliable triage system for early identification of subjects eligible for reperfusion therapy. It is also crucial to exclude intracranial haemorrhage and stroke-mimicking conditions before initiating therapy to avoid giving inappropriate or potentially life-threatening IV thrombolysis. An ideal triage system could potentially be used in prehospital settings to determine both immediate care (particularly in remote areas) and transfer arrangements to appropriate hospital facilities.

An increasing number of studies assessing the diagnostic performance of clinical assessment tools has been seen in recent years. A systematic review of stroke recognition instruments in suspected stroke patients was performed by Rudd et al. (2016) [4], and included studies that were published before 10 August 2015. The current review follows directly on from this date, and has been designed with the aim of answering the following questions:

1. What is the sensitivity and specificity of currently available clinical assessment tools for detecting subjects with ischaemic stroke due to LVO?
2. What is the sensitivity and specificity of currently available clinical assessment tools for diagnosing acute haemorrhagic stroke?
3. What is the sensitivity and specificity of currently available clinical assessment tools for differentiating between acute stroke and stroke-mimicking conditions?

## Methods

### Protocol and registration

The registered protocol can be accessed on PROSPERO, the international prospective register of systematic reviews:

https://www.crd.york.ac.uk/PROSPERO/display_record.php?RecordID=112492

### Eligibility criteria

Inclusion and exclusion criteria are presented in Table 1.

**Table 1:** Review inclusion and exclusion criteria

Domain	Inclusion	Exclusion
Study type	Comparative observational studies	Case reports
	Prospective observational studies	Selected case series
	Cohort studies	Literature review
	Unselected case series	Conference proceedings
		Full text unavailable
Participants	Human	Non-human subjects
	Adults	Exclusively paediatric patients Mixed paediatric and adult populations (where paediatric and adult groups are not possible to identify separately)
	Patients with ischaemic stroke (including patients with LVO), acute haemorrhagic stroke, and/or stroke-mimicking conditions and transient ischaemic attack	Exclusively patients with non-stroke conditions, such as sickle cell disease, arteriovenous malformation, traumatic brain injury, cerebral tumour, subarachnoid haemorrhage, vertigo etc.
	Sample size ≥300 participants [6]	Sample size < 300 participants
Setting	Prehospital and in-hospital	
Procedure	Use of a clinical assessment tool, including clinical scales, individual symptoms and signs designed for identification of patients with ischaemic stroke, including subjects with LVO, acute haemorrhagic stroke, and/or stroke-mimicking conditions	Exclusively assessing the diagnostic accuracy of a brain imaging modality for identification of patients with acute stroke and/or stroke-mimicking conditions
Aims/outcomes	Diagnostic accuracy of clinical tools designed for identification of patients with ischaemic stroke, including subjects with LVO, acute haemorrhagic stroke, and/or stroke mimicking conditions	Exclusively assessing the prognosis of functional disability caused by a stroke
	Diagnostic accuracy of clinical tools designed for differentiation between two main subtypes of stroke – ischaemic and haemorrhagic	Exclusively evaluating factors associated with misdiagnosis of stroke
		Exclusively diagnosing stroke patients with no assessment of clinical factors
Publication	2015–2018	Before January 2015
	In English	In languages other than English

### Information sources

A systematic search of the literature was conducted in October 2018, using a database-specific search strategy for each of the following electronic databases: *PubMed, EMBASE, Scopus* and *The Cochrane Library*.

### Search strategy

The search strategy included the following combination of multiple iterations of MeSH and keyword terms relating to each component of the research questions: intracranial hemorrhages, cerebral intraparenchymal hematoma, cerebrovascular apoplexy, brain infarction, acute stroke, brain ischemia, cerebrovascular occlusion, cerebral infarction, transient ischemic attack, “stroke mimic*”, prehospital emergency care, emergency care, scoring methods, neurologic signs and symptoms, differential diagnosis, neurologic examination, predictive value of tests, sensitivity and specificity, logistic models.

The search was restricted to human studies, English language, adult participants, and publication years 2015–2018. This restricted publication date range was chosen to perform an updated analysis of the data available. A systematic review by Rudd et al. (2016) included prospective studies and excluded retrospective studies, research within a known stroke population, tools that were exclusively used by ambulance dispatchers or with telecommunication systems [4]; however all of these were included in our systematic analysis.

### Study selection

Titles of studies retrieved using the search strategy were screened by one of the review authors to identify studies that potentially met the inclusion criteria outlined in Table 1. The abstracts of those potentially eligible studies were independently assessed for eligibility by three review team members. Any disagreements between them over the eligibility of particular studies were resolved through discussion with a fourth reviewer.

Eligible papers were tabulated and used in the qualitative synthesis. Studies which reported diagnostic accuracy values such as sensitivity, specificity, and positive and negative predictive values were included in the quantitative meta-analysis.

### Data collection process

A dedicated data extraction form was developed and used to collect relevant information from the included studies. The inclusion of information fields in the data collection form was guided by the review questions. The following components were assessed:

- study identification: name of the first author and publication year;
- setting for the application of the studied clinical tool: prehospital or in-hospital;
- inclusion/exclusion criteria for participants;
- sample size;
- name of the clinical assessment tool studied (where applicable);
- clinical information collected;
- background of personnel collecting and interpreting clinical information;
- diagnostic approach used to establish a final diagnosis;
- diagnostic accuracy values: true positive, true negative, false positive, false negative values, positive and negative predictive values, and/or positive and negative likelihood ratios, sensitivity and specificity.

As our analysis concerned only published data, no further data were sought from investigators.

### Risk of bias assessment in individual studies

Two review authors qualitatively assessed included studies for a risk of bias and concerns regarding their applicability for each of three domains: patient selection, index test, and flow and timing, in accordance with the QUADAS-2 Tool quality assessment system [5]. A table summarising risk of bias and applicability concerns was constructed.

### Data synthesis

The synthesis was performed in accordance with the Cochrane guidelines for diagnostic test accuracy reviews. The diagnostic accuracy data from each study were presented graphically by plotting sensitivities and specificities on a coupled column chart.

## Results

### Study selection

**Fig. 1:**
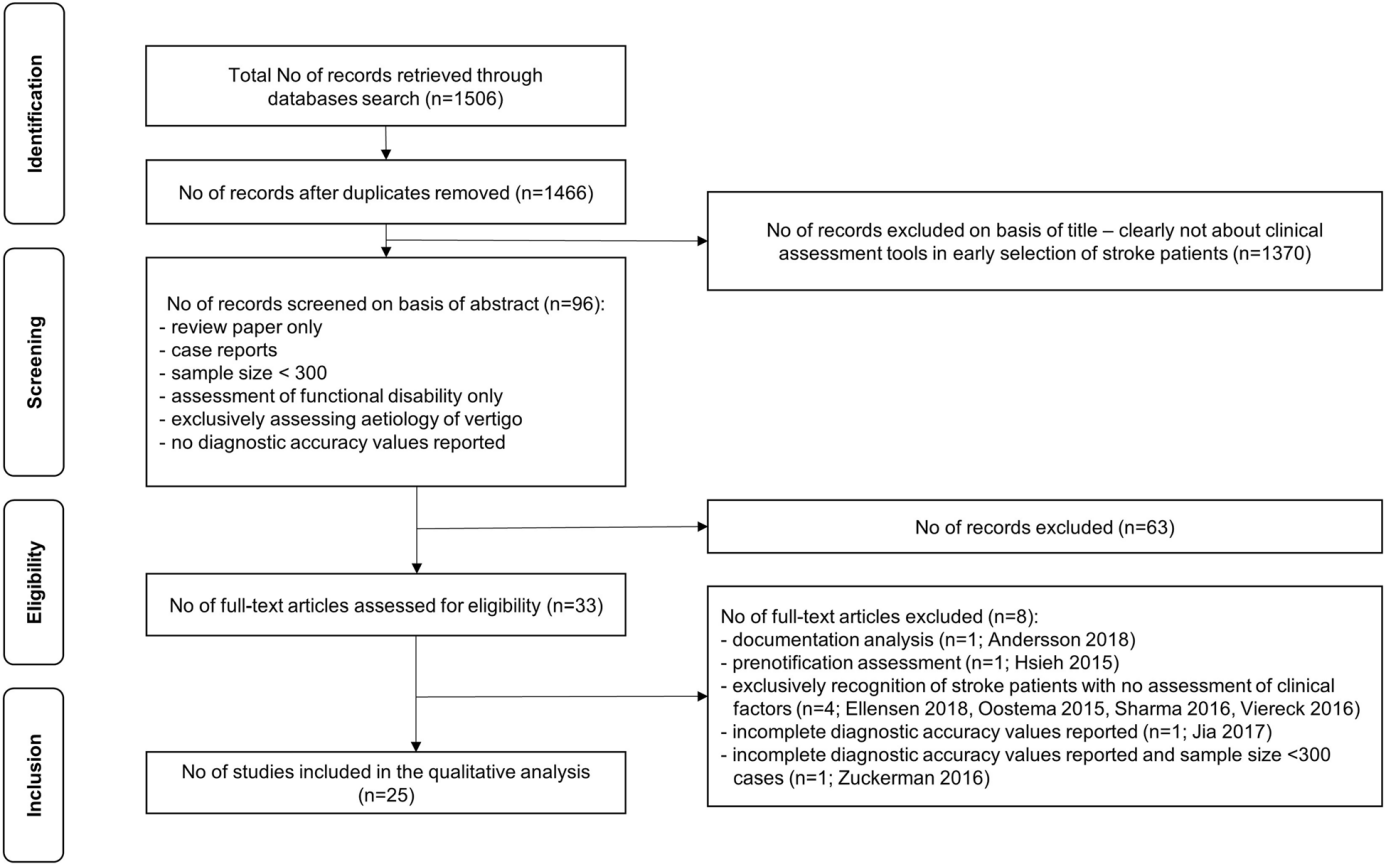
PRISMA flowchart. Outline of the study selection process using inclusion and exclusion criteria

The results of the study selection process are illustrated in Fig. 1.

### Study characteristics

The current review includes 25 new studies whereas adding to Rudd et al’s review (2016) [4], which included 18 primary studies out of the total number of 5622 references identified. The main characteristics of the studies included in the current review are presented in Additional file 1.

In total, 25,642 cases were assessed across the included studies published between 2015 and 2018. Participants were recruited in the prehospital setting, or upon presentation to the hospital, or both. Of the included studies, 16/25 (64%) were retrospective.

### Risk of bias assessment in individual studies

A summary of bias and applicability concerns is presented in Additional file 2.

All included studies recruited consecutive patients. Case-control methodology was avoided in all cases. Only studies with a high sample size of more than 300 participants according to Meader et al’s (2014) classification [6] were considered for inclusion to ensure greater reliability of the study results. All included studies were analysed against the adequate blinding criterion.

In 13/25 (52%) papers it was not specifically mentioned or was judged to be unclear whether the results of clinical assessment (the index test) were interpreted independently from those tests that were used to make a final diagnosis (the reference test) [7–19].

The approach for establishing the final diagnosis was described in all included studies. Hospital discharge diagnosis was referred to as the gold standard in six papers, brain or cerebral vessel imaging alone in 14 cases, and five papers used clinical assessment together with imaging to establish the definitive diagnosis.

### Diagnostic accuracy of clinical tools in selecting subjects with LVO

More than 20 different clinical assessment tools with optimal cut-offs for selecting subjects with ischaemic stroke due to LVO were analysed in this review (Table 2).

**Table 2:** Diagnostic accuracy values of clinical tools for selecting subjects with large vessel occlusion

Clinical assessment	Se	Sp	PPV	NPV	AUC
ACT-FAST [18]	0.85	0.93	0.53	0.99	0.90
aNIHSS ≥1 [27]	0.95	0.31	0.40	0.92	–
a-NIHSS item profile A ≥ 3 [37]	0.64	0.69	0.76	0.55	0.66
a-NIHSS item profile B ≥ 2 [37]	0.75	0.64	0.76	0.63	0.71
a-NIHSS item profile C ≥ 2 [37]	0.51	0.73	0.75	0.50	0.60
a-NIHSS item profile D ≥ 2 [37]	0.76	0.61	0.75	0.62	0.70
a-NIHSS item profile E ≥ 2 [37]	0.71	0.65	0.76	0.60	0.68
Aphasia; Neglect/gaze deviation [19]	0.91	0.70	0.60	0.94	0.77
Arm weakness [14]	0.96	0.41	0.18	0.99	0.68
Arm weakness; Leg weakness; Dysarthria [20]	0.92	0.44	0.28	0.96	–
Bernese score 1 ≥ 5 [37]	0.73	0.79	0.85	0.66	0.76
Bernese score 2 ≥ 2 [37]	0.71	0.80	0.84	0.64	0.74
Bernese score 3 ≥ 2 [37]	0.67	0.86	0.88	0.63	0.75
Bernese score 4 ≥ 1 [37]	0.67	0.83	0.86	0.63	0.74
Bernese score 5 ≥ 3 [37]	0.77	0.83	0.87	0.70	0.79
CPSS ≥1 [27]	0.96	0.24	0.38	0.93	–
CPSS ≥2 [14, 26]	0.56–0.92	0.81–0.85	0.40–0.65	0.78–0.99	0.75–0.78
CPSS1 ≥ 2 [37]	0.59	0.77	0.80	0.55	0.66
CPSS2 ≥ 2 [37]	0.73	0.71	0.79	0.63	0.72
CPSSS ≥1 [37, 38]	0.60–0.86	0.80–0.87	0.88	0.59	0.71
CPSSS ≥2 [9, 10, 24, 27, 38]	0.59–0.83	0.40–0.89	0.66–0.77	0.77–0.83	0.72–0.80
CPSSS ≥3 [38]	0.51	0.93	–	–	–
CPSSS =4 [38]	0.25	0.96	–	–	–
C-STAT [13]	0.54	0.91	0.45	0.93	0.79
C-STAT ≥2 [8, 17, 18]	0.47–0.85	0.68–0.85	0.35–0.40	0.88–0.98	0.65–0.85
DIRECT criteria [16]	0.74	0.92	0.43	0.98	0.91
EMSA ≥3 [8]	0.75	0.50	–	–	0.69
Expressive aphasia [14]	0.39	0.64	0.13	0.88	0.52
Facial weakness [14]	0.93	0.50	0.20	0.98	0.72
FAST = 3 [17]	0.84	0.44	0.32	0.90	0.53
FAST-ED [13]	0.62	0.84	0.40	0.94	0.84
FAST-ED ≥3 [26]	0.71	0.78	0.62	0.84	0.76
FAST-ED ≥4 [8, 18, 24, 26]	0.42–0.96	0.82–0.90	0.24–0.80	0.82–1.00	0.64–0.91
FPSS [13]	0.54	0.91	0.47	0.93	0.85
Gaze deviation [14]	0.84	0.73	0.30	0.97	0.79
G-FAST ≥3 [13, 17]	0.75–0.89	0.39–0.83	0.31–0.39	0.92–0.96	0.51–0.85
G-FAST =4 [13]	0.36	0.97	0.59	0.91	
LAMS ≥4 [9, 14, 18]	0.57–0.94	0.74–0.85	0.28–0.66	0.78–0.99	0.70–0.89
mNIHSS ≥5 [37]	0.78	0.76	0.85	0.69	0.78
mNIHSS ≥7 [27]	0.77	0.77	0.62	0.87	–
MPSS ≥3 [27]	0.84	0.65	0.54	0.89	–
Neglect [14]	0.88	0.69	0.28	0.98	0.79
Neglect/gaze deviation [14]	0.95	0.64	0.27	0.99	0.79
NIHSS ≥4 [14, 27]	0.93–0.99	0.24–0.46	0.15–0.46	0.93–0.99	0.62
NIHSS ≥5 [27]	0.90	0.54	0.49	0.92	–
NIHSS ≥6 [8, 14, 26, 27]	0.66–0.99	0.39–0.70	0.18–0.55	0.85–1.00	0.68–0.72
NIHSS ≥7 [27, 37]	0.81	0.72–0.77	0.59–0.84	0.72–0.89	0.79
NIHSS ≥8 [14, 24]	0.72–0.98	0.48–0.81	0.21–0.71	0.82–0.99	0.71
NIHSS ≥9 [8, 24, 27]	0.53–0.81	0.72–0.85	0.59–0.75	0.81–0.89	–
NIHSS ≥10 [14, 24, 26, 27, 39]	0.64–0.98	0.56–0.88	0.23–0.78	0.80–0.99	0.75–0.84
NIHSS ≥14 [27]	0.61	0.88	0.72	0.82	–
NIHSS-8 [13]	0.63	0.89	0.44	0.94	0.84
NIHSS-8 ≥ 8 [7]	0.81	0.75	0.52	0.92	0.82
NIHSS subitem – LoC (1a) [14]	0.23	0.81	0.14	0.88	0.52
NIHSS subitem – LoC (1b) [14]	0.60	0.56	0.16	0.91	0.58
NIHSS subitem – LoC (1c) [14]	0.49	0.68	0.17	0.91	0.59
NIHSS; Absence of prestroke handicap (mRS ≤ 2); Hemineglect; AF; Female sex; Total score cut-off ≥16 [39]	0.84	0.68–0.71	0.41–0.54	0.92–0.94	0.84
NIHSS symptom profile A or B [17]	0.76	0.65	0.40	0.90	0.68
OoH-NIHSS ≥1 [27]	0.96	0.24	0.38	0.93	–
PASS [13]	0.69	0.85	0.40	0.95	0.81
PASS ≥2 [9, 14, 17, 18, 24]	0.64–0.96	0.59–0.84	0.33–0.74	0.81–1.00	0.63–0.89
Pomona ≥1 [14]	0.98	0.50	0.21	0.99	0.74
Pomona ≥2 [14]	0.86	0.71	0.71	0.97	0.79
RACE ≥3 [37]	0.74	0.80	0.85	0.67	0.77
RACE ≥5 [8, 9, 16–18, 24, 26–28]	0.46–0.92	0.68–0.91	0.27–0.81	0.78–0.99	0.67–0.90
RACE V1 ≥ 4 [28]	0.85	0.56	–	–	0.81
RACE V2 ≥ 4 [28]	0.85	0.62	–	–	0.79
RACE V3 ≥ 3 [28]	0.89	0.48	–	–	0.78
RACE V4 ≥ 4 [28]	0.87	0.56	–	–	0.80
RACE V5 ≥ 4 [28]	0.83	0.57	–	–	0.79
RACE V6 ≥ 4 [28]	0.83	0.63	–	–	0.77
RACE V7 ≥ 3 [28]	0.87	0.51	–	–	0.76
Reduced level of consciousness with inability to answer questions; Leg weakness; Dysarthria; Gaze deviation [20]	0.96	0.39	0.27	0.98	–
Reduced level of consciousness with inability to answer questions; Facial weakness; Arm weakness; Sensation loss; Aphasia [20]	0.99	0.28	0.99	0.25	–
Reduced level of consciousness with inability to answer questions; Leg weakness; Neglect; Gaze deviation [20]	0.85	0.45	0.26	0.93	–
rNIHSS (profile A, B, C, D or E vs. profile F) [27]	0.83	0.61	0.51	0.88	–
ROSIER ≥4 [27]	0.79	0.76	0.61	0.88	–
sCPSSS (severe arm weakness, conjugate gaze deviation) ≥1 [38]	0.83	0.83			
sCPSSS ≥2 [38]	0.59	0.90			
sCPSSS =3 [38]	0.50	0.94			
sNIHSS-1 ≥ 1 [37]	0.63	0.80	0.83	0.59	0.70
sNIHSS-1 ≥ 2 [27]	0.66	0.81	0.63	0.83	
sNIHSS-5 ≥ 2 [37]	0.76	0.77	0.84	0.68	0.76
sNIHSS-5 ≥ 3 [24]	0.69	0.81	0.70	0.80	0.80
sNIHSS-5 ≥ 4 [27]	0.72	0.80	0.63	0.85	–
sNIHSS-8 ≥ 4 [37]	0.78	0.76	0.84	0.69	0.77
sNIHSS-8 ≥ 6 [24, 27]	0.64–0.77	0.78–0.88	0.63–0.78	0.79–0.88	0.82
sNIHSS-EMS ≥6 [24]	0.70	0.81	0.70	0.81	0.81
VAN [14]	0.95	0.56	0.23	0.99	0.77
Visual field defect [14]	0.88	0.75	0.33	0.98	0.82
3I-SS [9, 13]	0.42–0.50	0.92–0.93	0.45–0.77	0.77–0.92	0.71–0.78
3I-SS ≥1 [37]	0.73	0.78	0.83	0.65	0.75
3I-SS ≥2 [17]	0.65	0.72	0.42	0.87	0.71
3I-SS ≥4 [8, 24, 27]	0.19–0.40	0.94–0.95	0.74–0.85	0.71–0.74	0.65–0.80

Abbreviations: *AUC* area under the curve, *NPV* negative predictive value, *PPV* positive predictive value, *Se* sensitivity, *Sp* specificity

**Fig. 2:**
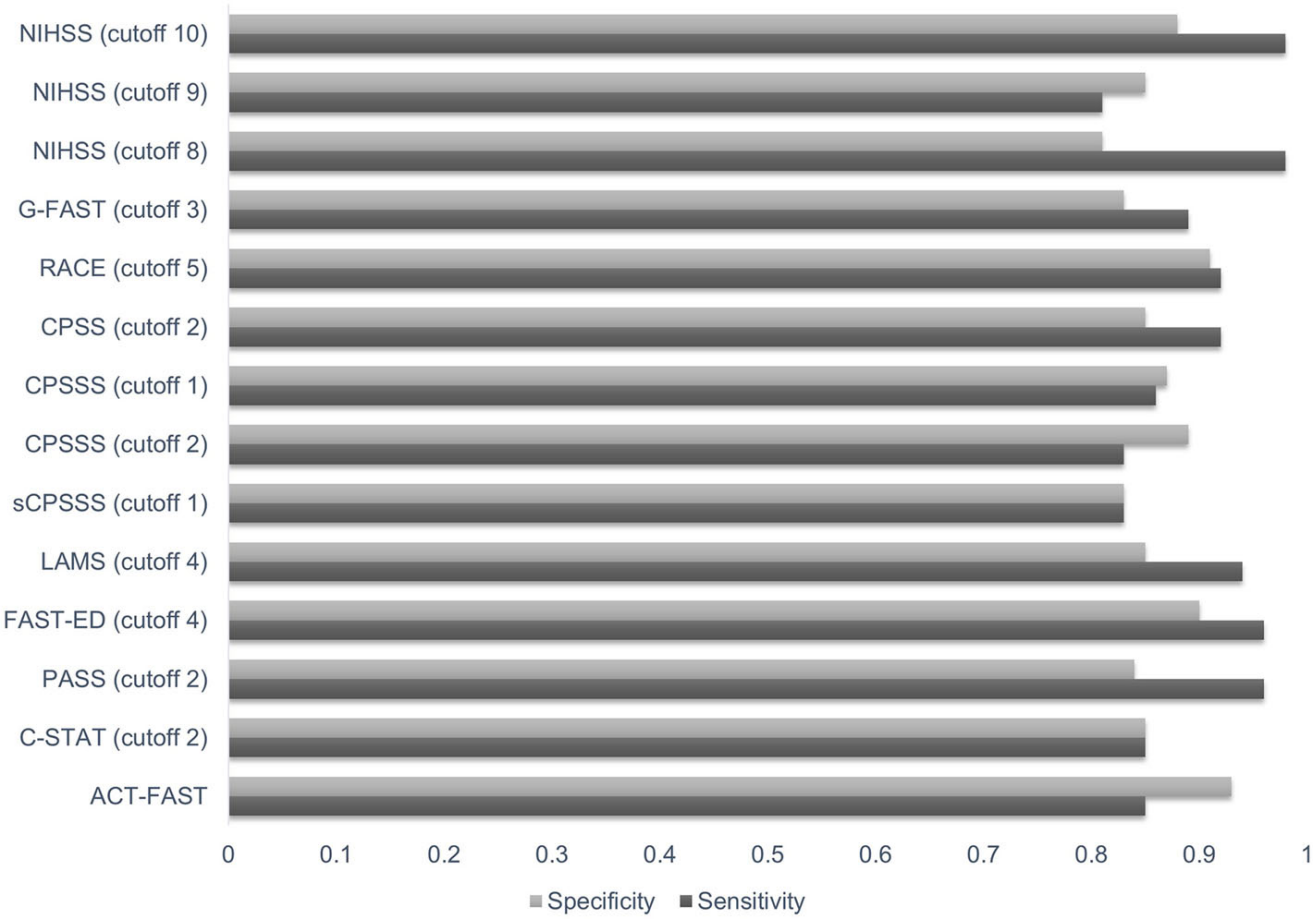
Bar chart. Sensitivity and specificity values across the clinical tools for selecting subjects with large vessel occlusion

Sensitivity values ranged from 23% (NIHSS subitem LoC 1a) to 99% (NIHSS≥4, NIHSS≥6, a combination of reduced level of consciousness with inability to answer questions, facial weakness, arm weakness, sensation loss, and aphasia). Specificity ranged from 24% (OoH-NIHSS≥1, CPSS≥1) to 97% (G-FAST = 4). For simplicity, only those tools showing both sensitivity and specificity values ≥80% (an arbitrarily chosen threshold) were selected to be plotted (Fig. 2).

It was suggested by Beume et al. (2018) [19] that cortical signs such as gaze deviation, aphasia or agnosia, and/or neglect were more accurate predictors of LVO than motor deficit alone (PPV 60%, NPV 94%). However, as demonstrated in Fig. 2, FAST-ED ≥ 4 (PPV 80%, NPV 100%), NIHSS≥10 (PPV 78%, NPV 99%), and RACE≥5 (PPV 81%, NP 99%) had the best diagnostic accuracy for selecting subjects with LVO. All three clinical assessment tools incorporate cortical signs as well as motor dysfunction.

The best sensitivity value of the combination of motor deficit and cortical signs incorporated into the Finnish Prehospital Stroke Scale (face drooping, limb weakness, speech difficulty, visual disturbance, and conjugate eye deviation) was for detection of proximal M1 occlusions (100%) and the lowest – for M2 and basilar artery – were 13 and 22%, respectively [13].

Moore et al. (2016) demonstrated that presence of all four components forming a combination of reduced consciousness level, lower limb weakness, dysarthria, and gaze deviation had sensitivity of 96% and specificity of 39% for LVO when compared with computed tomography angiography (CTA) [20]. Thus, those who do not have all four clinical features are less likely to have LVO, and therefore would not require CTA, decreasing the need for this test by about 32%. This approach might also contribute to decisions about immediate transfer to an endovascular centre for MT.

### Diagnostic accuracy of clinical tools in detecting acute haemorrhagic stroke

The paper by Jin et al. (2016) [21] was the single eligible study for the present review that aimed to distinguish between two main subtypes of stroke – ischaemic stroke and haemorrhage. A total of 1989 cases from the Chinese population with suspected first-ever acute stroke were analysed. They proposed a discriminant function model based on the following clinical assessment findings: age above 65 years, past medical history of diabetes (DM), atrial fibrillation (AF), systolic blood pressure (SBP) above 180 mmHg, and vomiting at onset. It has shown a higher sensitivity but lower specificity for selecting subjects with ischaemic stroke (42–75.7% and 63.3–93.6%, respectively). Diagnostic accuracy values for haemorrhage were the opposite of the above: low sensitivity with higher specificity (58.5–93.6% and 42–79.2%, respectively). It has also been suggested that a history of AF and DM were more likely to be associated with ischaemic stroke, whereas high SBP and younger age were associated with haemorrhage.

### Diagnostic accuracy of clinical tools for differentiating between acute stroke and stroke-mimicking conditions

**Fig. 3:**
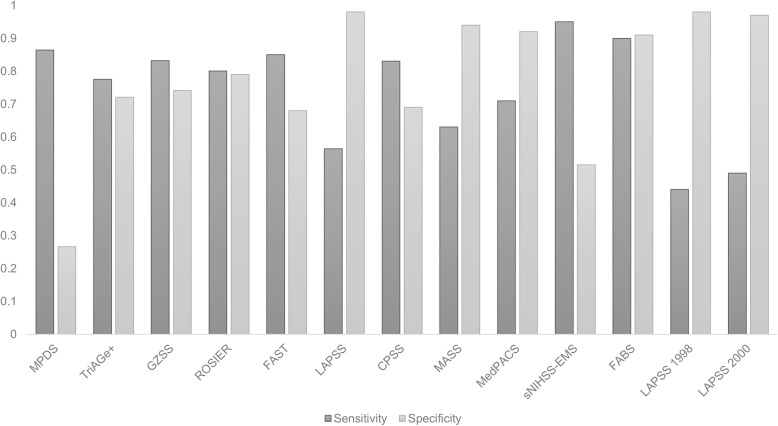
Bar chart. Sensitivity and specificity values of clinical tools for differentiating between acute stroke and stroke-mimicking conditions

There was a significant variation in diagnostic accuracy of tools designed for distinguishing between acute stroke and stroke mimics as shown in Fig. 3.

Sensitivity values varied from 44% (LAPSS 1998) to 91% (sNIHSS-EMS). Specificity ranged from 27% (MPDS) to 98% (LAPSS 1998) (Table 3). FABS showed the best diagnostic accuracy values with 90% sensitivity and 91% specificity [22].

**Table 3:** Diagnostic accuracy values of clinical tools for selecting subjects with acute stroke and stroke-mimicking conditions

Clinical tool	Target condition	Se	Sp	PPV	NPV	AUC
CPSS [15]	Acute stroke	0.83	0.69	0.50	0.91	–
FABS≥3 [22]	Stroke mimic	0.90	0.91	0.87	0.93	**–**
FAST [12, 15]	Acute stroke	0.76–0.85	0.64–0.68	0.50–0.93	0.30–0.92	0.70
GZSS≥1.5 [12]	Acute stroke	0.83	0.74	0.95	0.42	0.87
LAPSS [12]	Acute stroke	0.56	0.88	0.97	0.25	–
LAPSS 1998 [15]	Acute stroke	0.44	0.98	0.87	0.82	–
LAPSS 2000 [15]	Acute stroke	0.49	0.97	0.87	0.84	–
MASS [15]	Acute stroke	0.63	0.94	0.79	0.87	–
Med PACS [15]	Acute stroke	0.71	0.92	0.76	0.90	–
MPDS [23]	Acute stroke	0.86	0.27	0.20	0.90	**–**
ROSIER [12, 15]	Acute stroke	0.78–0.80	0.71–0.79	0.59–0.94	0.34–0.91	0.77
sNIHSS-EMS [24]	Acute stroke	0.91	0.52	0.43	0.93	–
TriAGe+ ≥ 10 [11]	Acute stroke	0.78	0.72	0.57	0.87	0.78

Abbreviations: *AUC* area under the curve, *NPV* negative predictive value, *PPV* positive predictive value, *Se* sensitivity, *Sp* specificity

The MPDS tool was developed to facilitate early identification of stroke or transient ischaemic attack by emergency medical dispatchers to enable early notification to receiving hospitals, and demonstrated satisfactory sensitivity of 86% but low specificity of 27% (PPV 20%, NPV 90%). Similarly, sNIHSS-EMS, which consisted of six NIHSS items selected as “suitable for prehospital use” [24] (level of consciousness, facial palsy, motor arm/leg, sensory, language and dysarthria), had the highest sensitivity value (91%) when compared with other clinical assessment tools but fairly low specificity (52%) (PPV 43%, NPV 93%). In contrast, LAPSS 1998 and LAPSS 2000 had the highest specificity (98 and 97%, respectively) but the lowest sensitivity (44 and 49%, respectively) values among the other tools [12, 15].

The FABS tool was developed for identification of subjects with stroke-mimicking conditions and negative brain CT findings in the emergency department. The total score is calculated based on the absent risk factors for stroke (AF, hypertension, advanced age) and presence of sensory disturbance with no motor deficit. FABS showed the best overall diagnostic accuracy values of 90% sensitivity and 91% specificity (PPV 87%, NPV 93%) [22].

## Discussion

A reliable triage system that could allow emergency transfer of patients eligible for MT directly to a regional centre with neurointerventional facilities following early IV thrombolysis (“drip and ship”) [25] could transform stroke care. The present systematic review attempted to evaluate the diagnostic accuracy of clinical assessment tools for (1) selecting subjects with ischaemic stroke due to LVO; (2) differentiating between two main subtypes of stroke – ischaemic stroke and haemorrhage, and (3) distinguishing between acute stroke cases and stroke mimics.

All reviewed studies had a minimum sample size of 300 consecutive participants [6] leading to good reliability of reported findings. There were however some limitations found in the included studies which were mainly related to unclear blinding of researchers interpreting the results of the index and reference tests, and discrepancy in the approach used to establish the gold standard.

As proposed by Beume et al. (2018) [19], cortical signs such as aphasia or neglect are more accurate predictors of LVO than motor deficit alone. However, a combination of signs suggestive of cortical involvement and motor deficit, for example, as assessed by FAST-ED, RACE or NIHSS scales, led to better diagnostic accuracy when compared to the performance of cortical signs alone as evaluated by the Pomona scale (Table 2).

Modest diagnostic accuracy was seen in clinical assessment tools aiming to distinguish between acute stroke and stroke mimics. The FABS tool which was designed specifically for detecting stroke mimics and included additional clinical information, such as atrial fibrillation compared to other well-established tools, for example, ROSIER, demonstrated high sensitivity and specificity rates of about 90% (PPV 87%, NPV 93%) [22]. Clinical assessment findings such as hypertension and younger age were indicative of haemorrhage, whereas a history of AF and DM were more likely to be associated with ischaemic stroke [21].

There are a few limitations of currently available tools that possibly prevent them from being widely accepted. First, their specificity rates for LVO remain quite low, which could potentially lead to inappropriate transportation of patients at high cost [27]. Second, many studies were designed in such a way that patients with haemorrhage and/or stroke-mimicking conditions were excluded, which therefore would preclude these clinical tools from being applied to prehospital settings [19].

An ideal clinical assessment tool would be a simple method that could be equally used in prehospital settings and in emergency department with high predictive values. It might be possible that a two-step approach using two different clinical assessment tools at the prehospital stage could be considered as an alternative option. The first step would be to select subjects with acute stroke who would benefit from reperfusion therapy and to exclude stroke mimicking conditions and acute intracranial haemorrhage. For this purpose, a tool with higher specificity should be considered, for example G-FAST [13]. This might allow prehospital thrombolysis to be offered to selected patients in remote areas in line with management of patients with S-T elevation myocardial infarction [29]. Thereafter, a decision on transferring subjects with suspected LVO to a specialised centre would be made on the basis of the clinical assessment score with high sensitivity value, such as NIHSS or a combination of clinical assessment findings as suggested by Moore et al. [14, 20, 27]. However, this approach requires further validation.

It might be beneficial to use an additional diagnostic tool in combination with clinical assessment that could provide valuable information and increase the accuracy of such a triage system. Transcranial Doppler ultrasound has been shown to detect occlusions in the major cerebral arteries with 68–100% sensitivity and 78–99% specificity [30–32]. It is a relatively inexpensive and readily portable diagnostic tool that takes on average not more than 15 min to complete an examination of the cerebral vessels [33], and can be used in prehospital settings, potentially with remote diagnostic support [34–36]. However, further assessment and validation of this proposed system is required.

## Conclusion

A wide range of clinical assessment tools for selecting subjects with acute stroke has been developed in recent years. Assessment of both cortical and motor function using RACE, FAST-ED or NIHSS demonstrated the best diagnostic accuracy values for selecting subjects with LVO. There were limited data on clinical tools that can be used to differentiate between acute ischaemia and haemorrhage. Diagnostic accuracy appeared to be modest for distinguishing between acute stroke and stroke mimics with optimal diagnostic performance demonstrated by the FABS tool. Further research is required to establish a novel prehospital triage system with possible application of a two-step clinical assessment or involvement of simple brain imaging, such as transcranial ultrasonography.

## Additional files

Additional file 1: Characteristics of included studies [7–24, 26–28, 37–40]. (DOCX 37 kb)

Additional file 2: Risk of bias and applicability concerns summary [7–24, 26–28, 37–40]. (DOCX 34 kb)

## Data Availability

Materials are available from the corresponding author upon request.

## Abbreviations

AF: atrial fibrillation
DM: diabetes mellitus
IV: intravenous
LVO: large vessel occlusion
MT: mechanical thrombectomy
NPV: negative predictive value
PPV: positive predictive value
SBP: systolic blood pressure
Se: sensitivity
Sp: specificity
