# Supplementary material for: Diagnostic accuracy of clinical tools for assessment of acute stroke: a systematic review
Source: BMC Emerg Med. 2019 Sep 4;19:49. doi: 10.1186/s12873-019-0262-1 (PMC6727516; doi:10.1186/s12873-019-0262-1)
Supplement: Supplementary file 1 — Characteristics of included studies [7–24, 26–28, 37–40]. (DOCX 37 kb) [file 12873_2019_262_MOESM1_ESM.docx]

Additional file 1. Characteristics of included studies

| Reference | Study type | Sample size | Clinical tool(s) | Target Condition | Application setting of the clinical tool | Gold standard |
| --- | --- | --- | --- | --- | --- | --- |
| Beume 2018 [19] | Retrospective | 543 | NIHSS items (hemiparesis; neglect/gaze deviation; aphasia) | LVO | Prehospital | Final records |
| Carrera 2017 [27] | Prospective | 341 (LVO n=71/341, 20%) | RACE  sRACE V1-V7 | LVO | Prehospital | NIHSS + transcranial ultrasound / CTA / MRA / Arteriography |
| Chen 2016 [40] | Retrospective | 1216 (posterior AIS n=100/1216, 8.2%) | A combination of clinical assessment findings | Posterior AIS | In-hospital | Discharge diagnosis |
| Clawson 2016 [23] | Retrospective | 1129 | MPDS | Stroke, TIA | Prehospital | Discharge diagnosis |
| Demeestere 2017 [7] | Retrospective | 551 (AIS n=381/551, 69%; LVO n=136/381, 36%) | NIHSS-8 | LVO | Prehospital | CTA |
| Goyal 2016 [22] | Prospective | 784 (stroke mimics n=321/784, 41%) | FABS | Stroke mimics | In-hospital | Clinical assessment, brain imaging (CT, MRI DWI, CTA, MRA, EEG), discharge diagnosis |
| Gropen 2018 [8] | Retrospective | 1663 (LVO n=171/1663, 10%) | EMSA  3I-SS  C-STAT  NIHSS  RACE  FAST-ED | LVO | Prehospital | Clinical assessment and brain imaging (CTA/MRA) |
| Hastrup 2016 [9] | Retrospective | 3127 | PASS  3I-SS  LAMS  RACE  CPSSS | LVO | Prehospital | CTA/MRA |
| Heldner 2016 [37] | Retrospective | 1085 | NIHSS  Bernese scores 1-5  RACE  3I-SS  sNIHSS-8 sNIHSS-5 sNIHSS-1 mNIHSS  a-NIHSS items profiles A–E  CPSS1  CPSS2  CPSSS | LVO | Prehospital | CTA/MRA |
| Jin 2016 [21] | Prospective | 1989 | Basic demographics and clinical characteristics | Stroke subtype (AIS vs ICH) | Prehospital | CT/MRI |
| Katz 2015 [10] | Prospective | 303 | CPSSS | LVO | Prehospital | CTA |
| Kummer 2016 [38] | Prospective | 751 (LVO n=80/751, 11%) | CPSSS | LVO | Prehospital | Neuroimaging (CTA/MRA/DSA) |
| Kuroda 2017 [11] | Prospective | 498 (stroke n=147/498, 30%) | TriAGe+  ABCD2 | Acute stroke | In-hospital | Neurology assessment, CT/MRI |
| Lima 2016 [26] | Prospective | 727 (LVO n=240/727, 33%) | FAST-ED  NIHSS  RACE  CPSS | LVO | Prehospital | Non-contrast CT, CTA |
| Mao 2016 [12] | Prospective | 416 | GZSS  ROSIER  FAST  LAPSS | Acute stroke | Emergency setting (prehospital and in-hospital?) | Clinical assessment, brain imaging (CT, MRI) |
| Moore 2016 [20] | Retrospective | 522 | Combinations of NIHSS criteria | LVO | In-hospital | CTA |
| Ollikainen 2018 [13] | Retrospective | 856 (AIS n= 462/856, 54%; ICH n=115/856, 13%; stroke mimics n=279/856, 33%) | FPSS  NIHSS-8  G-FAST  3I-SS  C-STAT  PASS  FAST-ED | Acute stroke, LVO | Prehospital | Non-contrast CT, CTA |
| Panichpisal 2018 [14] | Retrospective | 776 (LVO n=94/776, 12%) | Pomona Tool  Neglect/gaze deviation  NIHSS items  NIHSS  LAMS  CPSS  VAN  PASS | LVO | Prehospital and in-hospital | CTA/MRA |
| Purrucker 2015 [15] | Retrospective | 640 | CPSS  FAST  LAPSS 1998, 2000  MASS  Med PACS  ROSIER | Acute stroke | In-hospital | Discharge diagnosis |
| Purrucker 2017 [24] | Retrospective | 689 (stroke recognition cohort)  741 (LVO validation cohort) | sNIHSS-EMS  sNIHSS-8  sNIHSS-5  3I-SS  RACE  CPSSS  FAST-ED  PASS | Acute stroke  LVO | Prehospital | Discharge diagnosis, CTA |
| Rodríguez‐Pardo 2017 [16] | Retrospective | 317 | DIRECT | LVO | Prehospital | Neurological evaluation |
| Scheitz 2017 [17] | Retrospective | 3505 (LVO n=827/3505, 24%) | 3I-SS  RACE  C-STAT  PASS  NIHSS  FAST  G-FAST  NIHSS symptom profile A/B | LVO | Prehospital | Vessel imaging |
| Turc 2016 [27] | Retrospective | 1004 (LVO n=328/1004, 33%) | NIHSS  3I-SS  RACE  CPSSS  MPSS  mNIHSS  sNIHSS-1  sNIHSS-5  sNIHSS-8  aNIHSS  OoH-NIHSS  rNIHSS  profiles A-E vs. F  ROSIER | LVO | In-hospital | DWI, follow-up CT, MRA/CTA |
| Vanacker 2016 [39] | Retrospective | 1645 (LVO n=316/1645, 21%) | Integer score (NIHSS;  hemineglect;  female sex;  AF;  no history of stroke or stroke handicap)  NIHSS | LVO | In-hospital | CTA, MRA, MRI |
| Zhao 2018 [18] | Prospective | 565 | ACT-FAST  RACE  LAMS  FAST-ED  PASS  C-STAT | LVO | Prehospital | Stroke physician’s assessment |

Abbreviations: 3I-SS - 3-item stroke scale; aNIHSS – abbreviated National Institutes of Health Stroke Scale; CPSS - Cincinnati Prehospital Stroke Scale; CPSSS - Cincinnati Prehospital Stroke Severity Scale; C-STAT - the Cincinnati Stroke Triage Assessment Tool; DIRECT - the Direct Referral to Endovascular Center; EMSA - Emergency Medical Stroke Assessment; FAST-ED - Field Assessment Stroke Triage for Emergency Destination; FPSS - the Finnish Prehospital Stroke Scale; KPSS - Kurashiki Prehospital Stroke Scale; LAMS – Los Angeles Motor Scale; LAPSS - Los Angeles Prehospital Stroke Screen; MASS - Melbourne Ambulance Stroke Screen; Med PACS - Medic Prehospital Assessment for Code Stroke; mNIHSS – modified National Institutes of Health Stroke Scale; MPDS - Medical Priority Dispatch System; MPSS - Maria Prehospital Stroke Scale; NIHSS - National Institutes of Health Stroke Scale; PASS - Prehospital Acute Stroke Severity Scale; RACE - Rapid Arterial Occlusion Evaluation; rNIHSS – retrospective National Institutes of Health Stroke Scale; ROSIER - Recognition of Stroke in the Emergency Room; sNIHSS-EMS - shortened NIHSS for emergency medical services; sRACE – simplified RACE; VAN – the Vision Aphasia and Neglect Scale.
