# Supplementary material for: Diagnostic accuracy of clinical tools for assessment of acute stroke: a systematic review
Source: BMC Emerg Med. 2019 Sep 4;19:49. doi: 10.1186/s12873-019-0262-1 (PMC6727516; doi:10.1186/s12873-019-0262-1)
Supplement: Supplementary file 2 — Risk of bias and applicability concerns summary [7–24, 26–28, 37–40]. (DOCX 34 kb) [file 12873_2019_262_MOESM2_ESM.docx]

Additional file 2. Risk of bias and applicability concerns summary

|  | **Risk of bias** | | | | | **Applicability concerns** | |
| --- | --- | --- | --- | --- | --- | --- | --- |
|  | **Patient selection** | **Index test** | | **Flow and timing** | | **Patient selection** | **Index test** |
| Beume 2018 [19] |  |  | |  | |  |  |
| Carrera 2017 [27] |  |  | |  | |  |  |
| Chen 2016 [40] |  |  | |  | |  |  |
| Clawson 2016 [23] |  |  | |  | |  |  |
| Demeestere 2017 [7] |  |  | |  | |  |  |
| Goyal 2016 [22] |  |  | |  | |  |  |
| Gropen 2018 [8] |  |  | |  | |  |  |
| Hastrup 2016 [9] |  |  | |  | |  |  |
| Heldner 2016 [37] |  |  | |  | |  |  |
| Jin 2016 [21] |  |  | |  | |  |  |
| Katz 2015 [10] |  |  | |  | |  |  |
| Kummer 2016 [38] |  |  | |  | |  |  |
| Kuroda 2017 [11] |  |  | |  | |  |  |
| Lima 2016 [26] |  |  | |  | |  |  |
| Mao 2016 [12] |  |  | |  | |  |  |
| Moore 2016 [20] |  |  | |  | |  |  |
| Ollikainen 2018 [13] |  |  | |  | |  |  |
| Panichpisal 2018 [14] |  |  | |  | |  |  |
| Purrucker 2015 [15] |  |  | |  | |  |  |
| Purrucker 2017 [24] |  |  | |  | |  |  |
| Rodríguez‐Pardo 2017 [16] |  |  | |  | |  |  |
| Scheitz 2017 [17] |  |  | |  | |  |  |
| Turc 2016 [27] |  |  | |  | |  |  |
| Vanacker 2016 [39] |  |  | |  | |  |  |
| Zhao 2018 [18] |  |  | |  | |  |  |
|  | | | High | | Unclear | | Low |
